# Co‐occurrence of *JAK2* V617F‐mutated essential thrombocythemia and chronic lymphocytic leukemia harboring der(8;17)(q10;q10)

**DOI:** 10.1002/cnr2.1658

**Published:** 2022-06-17

**Authors:** Masahiro Manabe, Nao Tanizawa, Satoru Nanno, Yuuji Hagiwara, Reiko Asada, Ki‐Ryang Koh

**Affiliations:** ^1^ Department of Hematology Osaka General Hospital of West Japan Railway Company Osaka Japan; ^2^ Hematology, Graduate School of Medicine Osaka City University Osaka Japan; ^3^ Department of Clinical Laboratory Osaka General Hospital of West Japan Railway Company Osaka Japan

**Keywords:** cytogenetics, hematological cancer, pathology

## Abstract

**Background and Case:**

We herein present a case of the co‐occurrence of *JAK2*‐mutated essential thrombocythemia (ET) with chronic lymphocytic leukemia (CLL) harboring the recurrent and rare whole‐arm translocation, der(8;17)(q10;q10). The co‐existence of lymphoproliferative neoplasms and myeloproliferative neoplasms is suggested to be a rare event. Under this condition, the lymphoproliferative disorder presents a clinically indolent course with a low‐risk biological profile. However, the present case showed aggressive disease progression, reflecting a poor prognostic factor; that is, the loss of 17p caused by the whole‐arm der(8;17)(q10;q10) translocation.

**Conclusion:**

The present case report emphasizes the importance of considering the involvement of a genetically poor prognostic factor, regardless of the co‐occurrence of CLL and ET.

## 1 INTRODUCTION

Whole‐arm translocations are considered to be rare in hematological malignancies.^1^ der(8;17)(q10;q10) is a recurrent and rare chromosomal aberration associated with lymphoid neoplasms.^1^, ^2^, ^3^, ^4^, ^5^, ^6^ The co‐existence of lymphoproliferative neoplasms and myeloproliferative neoplasms is suggested to be a rare event, and the lymphoproliferative disease demonstrates a clinically indolent course with a low‐risk biological profile.^7^, ^8^ We herein report a case of the co‐occurrence of Janus kinase 2 (*JAK2*)‐mutated essential thrombocythemia (ET) and chronic lymphocytic leukemia (CLL) harboring der(8;17)(q10;q10), which resulted in the loss of 17p, considered to be a poor prognostic factor, and showed steady progression.

### 1.1 Case presentation

**FIGURE 1:**
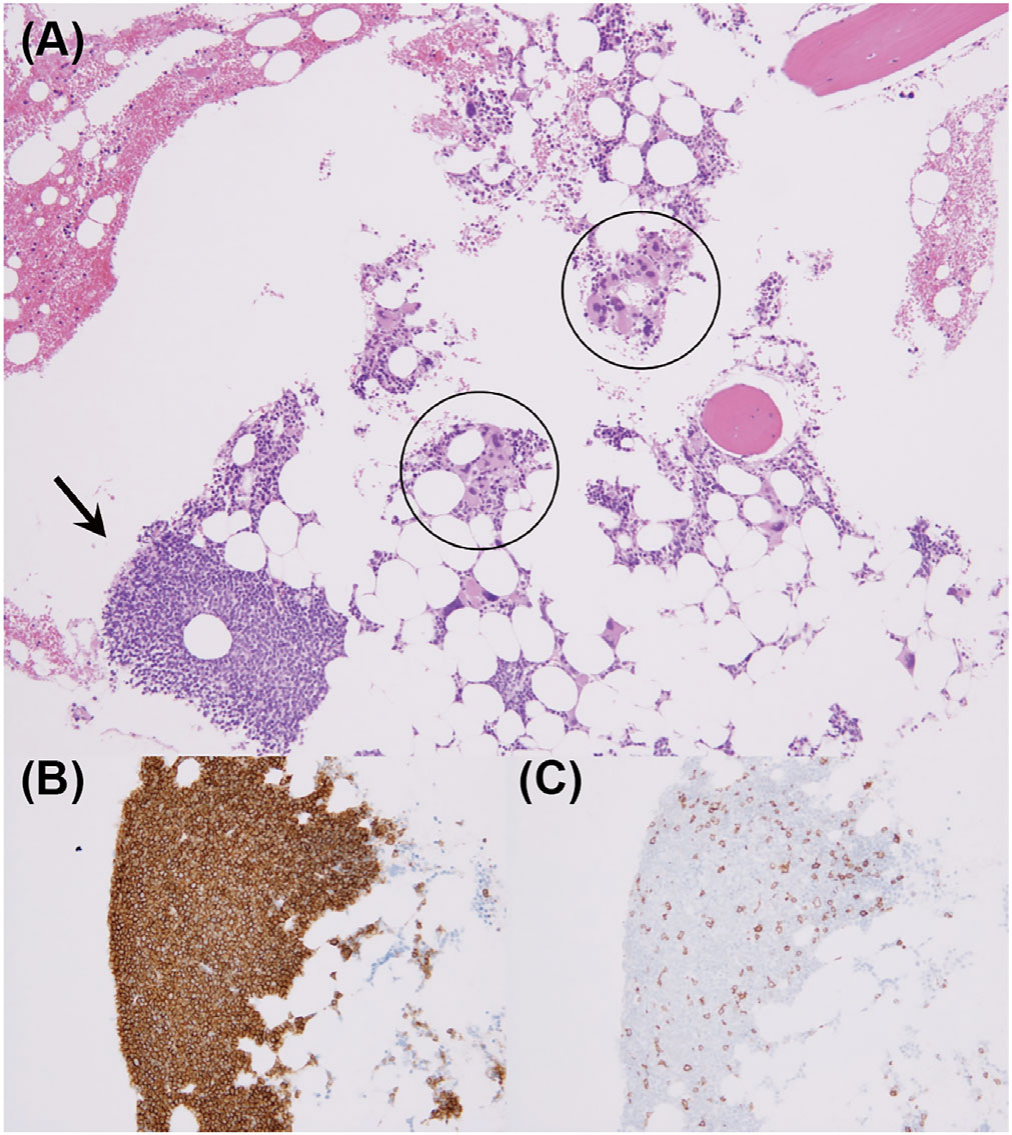
Pathological images of bone marrow biopsy. (A) Proliferation of megakaryocytes (circles) and clusters of small lymphocytes (arrow) were observed (hematoxylin and eosin staining, ×100). (B, C) Immunohistochemistry demonstrated that lymphocytes were positive for CD23 (B, ×200) and aberrantly positive for CD5 (C, ×200).

**FIGURE 2:**
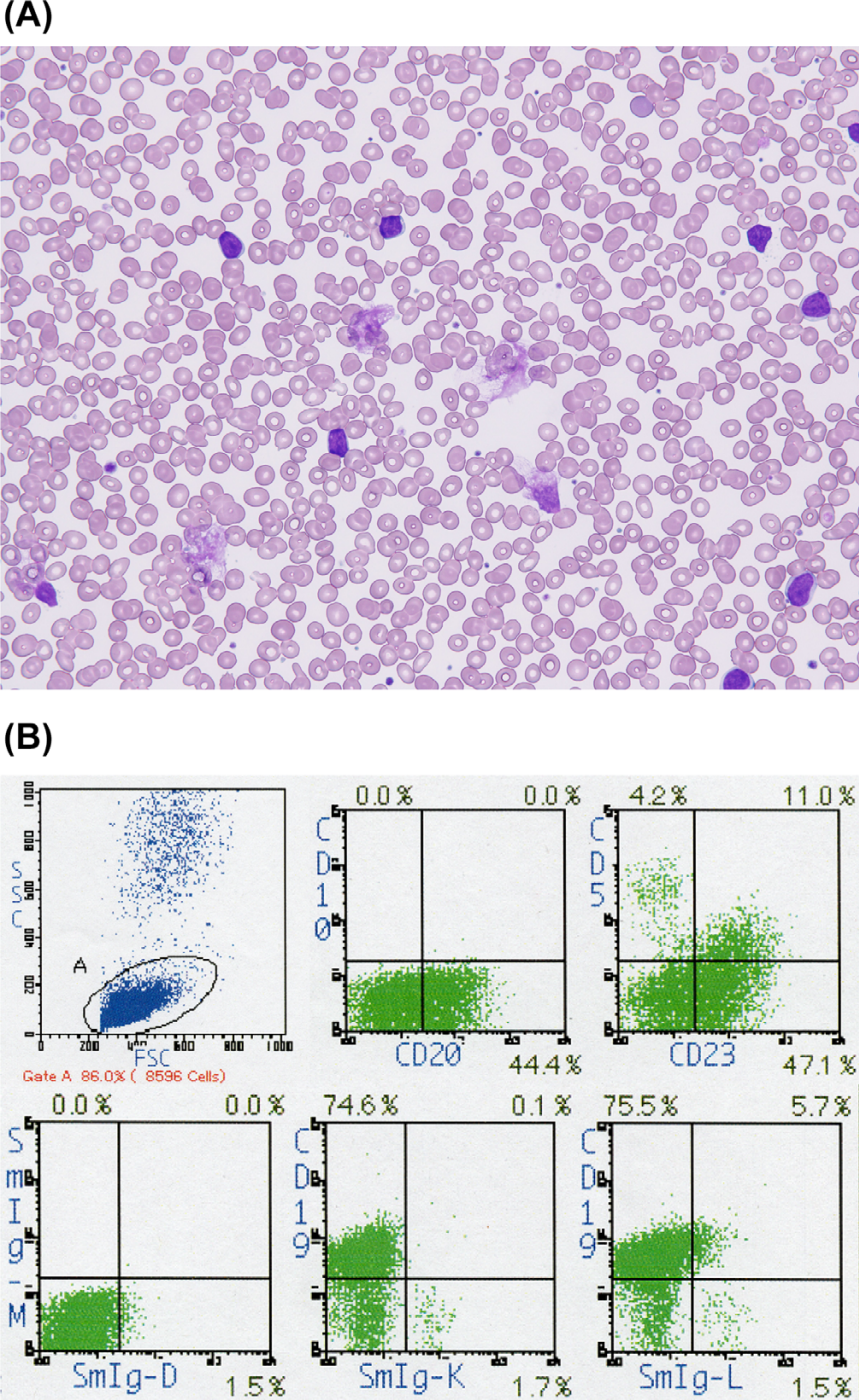
(A) Peripheral blood smear at the diagnosis of CLL (May‐Giemsa staining, ×400). (B) Flow cytometric analysis of peripheral blood cells by SSC/FSC gating. The corresponding cell percentage demonstrated by the gate is 86.0%. The results of two‐color analyses with CD10 and CD20, CD5 and CD23, surface (s‐)IgM and s‐IgD, CD19 and s‐Igκ, and CD19 and s‐Igλ for the gated cells are shown. Corresponding cell percentages in each fraction are indicated. The gated cells are positive for CD20, CD23, and CD19 and weakly positive for CD5. Strong positivity for surface immunoglobulin was not detected.

**FIGURE 3:**
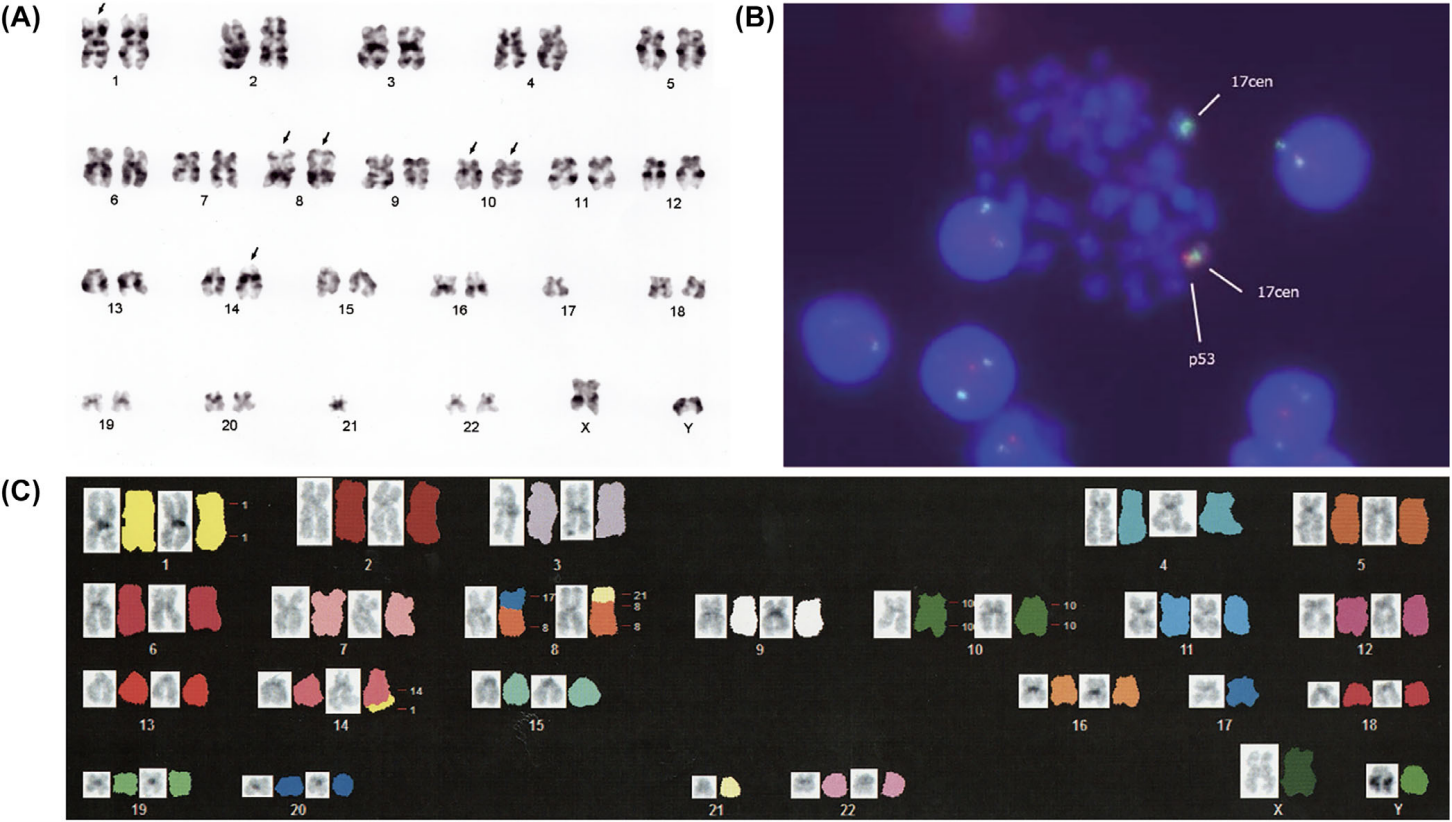
(A) G‐banded karyogram obtained in the present case. The arrows indicate derivative chromosomes. (B) FISH image showing the loss of one copy of the *TP53* gene. Red signal, the p53 probe; green signal, chromosome 17 centromere. (C) Spectral karyotyping of the metaphase spread after a spectrum‐based classification (left, counterstained with 4′,6‐diamino‐2‐phenylindole dihydrochloride; right, SKY).

A 74‐year‐old male was referred to our hospital (Osaka General Hospital of West Japan Railway Company, Osaka, Japan) with thrombocythemia in September 2015. His medical history included hypertension, which was being treated with oral amlodipine besilate. Although a physical examination showed mild splenomegaly, palpable lymph nodes were absent. The initial complete blood count test showed a white blood cell (WBC) count of 12.2 × 10^9^/L (reference range, 3.4–8.4 × 10^9^/L) with 73.5% neutrophils, 2.5% eosinophils, 6.5% basophils, 2.5% monocytes, and 15% lymphocytes. The concentration of hemoglobin was 13.2 g/dL (reference range, 14.0–18.0 g/dl) and the platelet count was 1224 × 10^9^/L (reference range, 130–320 × 10^9^/L). A polymerase chain reaction revealed the *JAK2*‐V617F mutation in peripheral blood cells. Bone marrow biopsy showed the proliferation of megakaryocytes and clusters of small lymphocytes (Figure 1). Immunohistochemical staining revealed that small lymphocytes were positive for CD23 and CD79a, aberrantly positive for CD5, and negative for cyclin D1. A chromosomal study of bone marrow cells showed a normal karyotype of 46,XY[20]. Based on these findings, the patient was diagnosed with the co‐occurrence of ET and monoclonal B‐cell lymphocytosis (MBL). Although he was treated with hydroxyurea (500 mg per day) and aspirin for ET, only watchful waiting was performed for MBL because a treatment intervention was not indicated at this point. In November 2017, a gradual increase in the WBC count was noted. Laboratory data included a WBC count of 23.6 × 10^9^/L with 49% lymphoid cells, a hemoglobin concentration of 10.8 g/dL, and platelet count of 294 × 10^9^/L. In a surface marker analysis, lymphoid cells in peripheral blood showed CD19, CD20, CD23, and CD5 (weak) expression (Figure 2). A follow‐up cytogenetic analysis revealed the following abnormal karyotype: 44,XY,t(1;14)(p36.1;q32),der(8)t(8;21)(p21;q?),der(8;17)(q10;q10),del(10)(p13) × 2,‐21 [3]/46,XY[17]. In addition, a fluorescent in situ hybridization analysis showed the loss of the p53 signal in 94% of cells (Figure 3). Therefore, the patient was diagnosed with CLL that evolved from MBL. At this point, oral hydroxyurea was discontinued due to anemia. Leukocytosis and anemia reflecting the progression of CLL gradually progressed in April 2018 (WBC, 28.7 × 10^9^/L; hemoglobin, 9.3 g/dl). Hemolysis was not detected. Although we proposed the initiation of ibrutinib monotherapy because anemia was an indication for treatment, the patient did not wish to be treated for financial reasons. Only follow‐up surveillance was continued; however, lymphocytosis became more prominent in April 2019 (WBC, 71.8 × 10^9^/L; lymphocytes, 83%), and the patient eventually died of pneumonia.

## 2 DISCUSSION

Regarding cytogenetic abnormalities among CLL, an abnormality of chromosome 17 is the most frequent in cases involving complex karyotypes.^9^ On the other hand, only seven cases of mature lymphoproliferative disorders (six of CLL^1^, ^2^, ^3^, ^4^, ^5^ and one of Waldenström's macroglobulinemia^6^) involving the der(8;17)(q10;q10) translocation have been reported in the literature; therefore, this translocation is considered to be recurrent and rare. Furthermore, it was impossible to establish the prognostic significance of this translocation on the prognosis of the patient because the majority of previous case reports mainly focused on the chromosomal abnormality itself or described a case series. Among CLL cases, the loss of 17p is associated with a poor prognosis and *TP53* mutations. der(8;17)(q10;q10) results in the loss of 8p and 17p due to the unbalanced nature of the translocation. Besides the simple deletion of 17p, the unbalanced translocation resulted in the loss of 17p, such as dic(17;18)(p11.2;p11.2),^10^ which may correlate with a poorer outcome. Regarding the abnormality in 14q32 in the present case, a recent study indicated that chromosome 14q32 rearrangements/translocations involving the immunoglobulin heavy chain (*IGH*) were associated with an intermediate‐adverse outcome.^11^ The co‐existence of lymphoproliferative neoplasms and myeloproliferative neoplasms is suggested to be a rare event. Under this condition, the lymphoproliferative disorder presents a clinically indolent course with a low‐risk biological profile.^7^, ^8^ However, regardless of the co‐occurrence of CLL and ET, a genetically poor prognostic factor was considered to be strongly involved.

## 3 CONCLUSION

The co‐occurrence of myeloproliferative neoplasms and CLL rarely occurs and presents an indolent clinical course. However, clinicians need to be aware that chromosomal abnormalities with a poor prognosis have a strong negative impact on the prognosis of patients.

## AUTHOR CONTRIBUTIONS

**Masahiro Manabe:** Conceptualization (lead); data curation (lead); formal analysis (lead); investigation (lead); methodology (lead); project administration (lead); resources (lead); writing – original draft (lead); writing – review and editing (lead). **Nao Tanizawa:** Investigation (equal); methodology (equal); writing – review and editing (equal). **Satoru Nanno:** Investigation (equal); methodology (equal); writing – review and editing (equal). **Yuuji Hagiwara:** Data curation (equal); investigation (equal); methodology (equal); writing – review and editing (equal). **Reiko Asada:** Data curation (equal); investigation (equal); methodology (equal); writing – review and editing (equal). **Ki‐Ryang Koh:** Investigation (equal); methodology (equal); writing – review and editing (equal).

## CONFLICT OF INTEREST

No conflicts of interest.

## ETHICS STATEMENT

Written informed consent was obtained from the patient for the publication of case details and the use of images.

## Data Availability

Data sharing is not applicable to this article as no new data were created or analyzed in this study.
